# A multiprofessional perspective on the principal barriers to universal health coverage and universal access to health in extremely poor territories: the contributions of nursing^1^

**DOI:** 10.1590/1518-8345.1042.2688

**Published:** 2016-05-03

**Authors:** Viviane Helena de França, Celina Maria Modena, Ulisses Eugenio Cavalcanti Confalonieri

**Affiliations:** 2 PhD, RN, Hospital Público Regional Prefeito Osvaldo Rezende Franco, Betim, MG, Brazil; 3 PhD, Researcher, Centro de Pesquisas René Rachou, Fundação Oswaldo Cruz (Fiocruz), Belo Horizonte, MG, Brazil

**Keywords:** Right to Heath, Poverty, Public Policy, Unified Health System, Primary Health Care, Community Health Nursing

## Abstract

**Objective:**

to investigate the knowledge of managers and health professionals, social workers and education professionals regarding the principal barriers to universal health coverage and universal access to health on the part of the extremely poor population; and to point to the contributions made by nursing for the promotion of this right.

**Method:**

a qualitative study whose reference was, for ensuring the right to health, the reorientation of the Brazilian Unified Health System (SUS) towards universal coverage and access in these territories. Interviews were held with 27 members of the multi-professional team of a municipality with high social vulnerability. The data were worked on using thematic content analysis.

**Results:**

the following were ascertained as the principal barriers to universal health coverage and access to health: failures in the expansion and strengthening of the services; absence of diagnosis of the priority demands; shortage of technology, equipment, and material and human resources; poor local infrastructure; and actions with low resolutive power and absence of interdepartmental policies. Within the multi-professional team, nursing acts in the SUS in unique health actions and social practices in these territories, presenting an in-depth perspective on this harsh reality, being able to contribute with indispensable support for confronting these disparities in universal health coverage and universal access to health.

**Conclusion:**

nursing's in-depth understanding regarding these barriers is essential for encouraging the processes reorienting the SUS, geared towards equality in the right to health.

## Introduction

In theory, the organization of the health services in Brazil is based on the Federal Constitution of 1998 and on the organic laws of the Unified Health System (SUS). For universal health coverage and universal access to health, in the municipalities, the following are stipulated by the SUS: financial investments, resources, and tripartite management processes, respecting the federal single management and decentralized municipal management, in order to prioritize the ensuring of attendance to the population's needs and their local requirements, taking into account the geographical, biological, social, economic and cultural adversities for care which is comprehensive, fair and inclusive^(^ 1 ^)^.

The availability or absence of equipment and devices which mediate universal health coverage and access to health in the municipalities is associated with various public services, health services, social work services, education, basic sanitation, productive inclusion and urban infrastructure, which can configure - or not - scenarios which are propitious for actions preventing disease and promoting health, treatment, rehabilitation and protection, favoring situations which can either reproduce or repair the context of poverty; and contribute - or not - to these groups gradually increasing or reducing their distance from achieving justice and citizenship^(^ 1 ^-^ 2 ^)^.

Health is a social, physical, biological, psychic, cultural and affective good, which is manifested as a condition of positive well-being when its achievement is mediated through the offering of easily-accessed equipment, facilities and devices, strengthening it in the face of life's challenges. The processes of expansion and broadening in the offering of tools which are compatible with meeting the population's health requirements require, therefore, public policies and actions which are coherent and legitimate for each context. These must be sustainable if they are to progressively meet the population's needs, using efficacious management processes, sufficient financial resources, and a professional team which has been empowered to eliminate the barriers to access to attendance and care and to integral health with fairness. In this regard, the strengthening of the interdepartmental actions is an essential effort^(^ 2 ^)^.

Resolution CP 53.14, of 2014, of the Pan-American Health Organization (PAHO), presents the essential requirements for encouraging actions preventing disease, and promoting health, treatment, rehabilitation, protection, and the construction of conditions which are favorable to the assistance and integral care of the population, in particular those groups in social vulnerability, and to create opportunities which ensure them the right to universal health coverage and to universal access to health. In order to analyze these groups' living conditions, it is not enough to identify the causes and consequences which act in this context, reinforcing the processes of inequality and social exclusion. It is necessary to reflect on the reality in these territories, aiming for a society which is more just and righteous, and to ask the question: inequality in what?^(3).^

In addressing the mismatches present in extremely poor territories, and seeking responses to the main problems and gaps in meeting these subjects' needs, the authors adopted the PAHO resolutions providing information on the principles and guidelines which are considered pillars for the planning and execution of public policies in favor of legitimate and sustainable actions capable of guaranteeing universal health coverage and universal access to health for the population of Latin America, in particular, for the vulnerable groups. 

Among the world's population - 7.2 billion, in 2015 - 836 million were considered extremely poor, surviving on less than $1.25 per day^(^ 4 ^)^. In Latin America, social inequality is high, as is the proportion of the population which is poor, with 29% below the poverty line, 30% without access to health for financial reasons, and 21% unable to seek attendance due to geographical barriers^(^ 5 ^)^ **_._** In Brazil, 16.27 million people are poor (8.6%), and the eradication of poverty is a major socioeconomic challenge. The *Poverty Line*, in Brazil, defines extreme poverty as family income *per capita* less than or equal to R$77.00 (seventy-seven *reais*) per month^(^ 6 ^)^. 

In order to define the degree of poverty, one can use unidimensional or multidimensional criteria which are capable of measuring the attendance to these subjects' basic needs. The multidimensional measuring is part of the criteria of privation of income, crucial aspects such as the lack of, or poor access to, the attendance of basic needs, material resources, goods, services, and social support. In the scenarios of extreme poverty, the access to integral healthcare facilitates the achieving of other social rights such as access to education, nutrition, basic sanitation, housing, and social and productive inclusion^(^ 7 ^)^.

In the big cities, extreme poverty is surrounded by a ring of disadvantages, acting as geographical, environmental, social, economic, and affective barriers which are difficult to overcome. Examples of these disadvantages are the prevalence of neglected diseases, mainly affecting families with worse living conditions, and contributing to the low level of social development found in some regions. These diseases reinforce these subjects' imprisonment in a repetitive cycle of disadvantages, marked by the absence of health or perspectives for work or social inclusion, with severe financial consequences^(^ 8 ^)^.

The guidelines of the Pan-American Health Organization, in line with the Unified Health System (SUS), can strengthen the resolutive capacity of the health services to meet the needs of families in extreme poverty in Brazil, overcoming the barriers to universal health coverage and universal access to health. Primary Health Care (APS) and the Family Health Strategy (ESF) have as their goal to promote health, holistic care, and initial access to this right in the SUS. Nursing is an area of health, management and care with great relevance in this context. The multi-professional team which acts in the public services, in particular nurses, can contribute to the reflection regarding problems of universal health coverage and universal access to health in these territories. This study aimed to investigate the knowledge of managers and health professionals, social workers, and educational professionals regarding the principal barriers to universal health coverage and universal access to health for the population in extreme poverty, and to point to the contributions which may be made by nursing for promoting this right.

## Method

This is exploratory-descriptive qualitative research, seeking to identify the meanings attributed to the barriers present to universal health coverage and universal access to health in the ambit of the SUS, in extremely poor territories. 

The study scenario is one of the 37 municipalities which make up the Metropolitan Region of Belo Horizonte (RMBH), which was selected as a result of the high incidence of poverty among its inhabitants, and because it is characterized by its shortage of public services, spatial segregation, and social exclusion. Its growth results from the logic of the center-periphery expansion of RMBH, amply defined by the already-established processes of immigration, with the concentration of a large number of inhabitants characterized by low income and low educational level. In the region where these families live, historically, there has been poor organization of urban infrastructure, with a large number of areas where people have built illegally and areas where people squat illegally. These aspects contribute to the vulnerability and social disadvantages of these extremely poor families in the region, due to the absence of an economic base capable of absorbing part of this workforce, associated with the presence of a prison complex, made up of five institutions, favoring a misrepresented and prejudiced view of the population

In order to collect the variety of information on the life context and health actions undertaken with families in extreme poverty in this region, and the local barriers to universal health coverage and universal access to health, the researchers chose to interview key informants from the municipality's multi-professional team from the areas of health, social work and education, so as to investigate this context in depth. Interviews were held with managers and professionals who work in planning and executing public policies at a central and regional level in these territories of extreme poverty. In order to select these subjects, 27 informants were invited. These participated in the research after signing the Terms of Free and Informed Consent (TFIC). The number of subjects was defined by the criteria of theoretical saturation, proceeding or not with the interviews according to the absence or presence of new elements which made it possible to deepen the analysis and theoretical understanding regarding the meanings. 

A semistructured script was previously validated, addressing the following themes: health, quality of life, and the principal problems experienced by the families in extreme poverty; public services available for meeting their needs; public health policies and interdepartmental policies in place in the region; the effects on their quality of life and health; and suggestions for improvements for ensuring the rights to health and gains in quality of life, in this context. 

The data were worked on by applying Thematic Content Analysis, with systematic procedures for organizing, codifying and categorizing the ideas, so as to indicate significant qualitative indicators on the phenomenon. As a result, the interviews were read exhaustively and in depth, based on the reference of the strategic lines proposed by PAHO in order to transform the SUS in relation to universal health coverage and universal access to health. 

**Figure 1:**
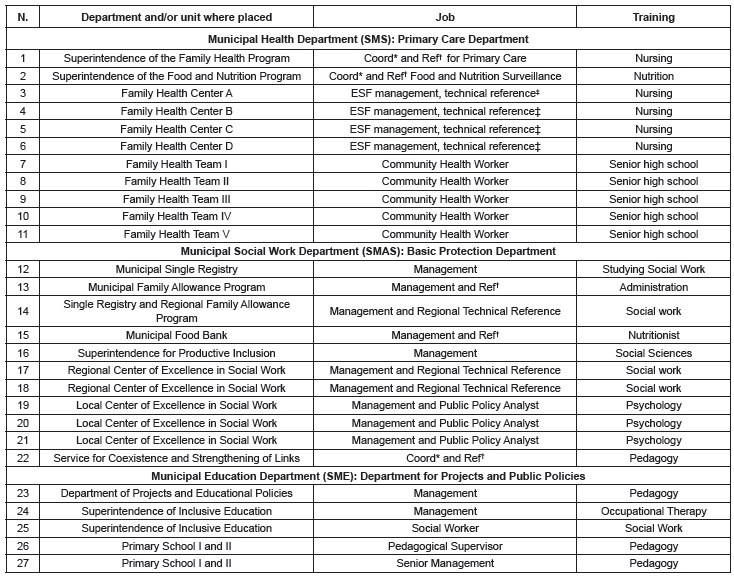
- The multi-professional team of the Municipal Health Department (SMS), Social Work Department (SMAS), Education Department (SME); by Department or Center where placed; Job and Training, in a municipality of the Metropolitan Region of Belo Horizonte, State of Minas Gerais (MG), Brazil

This study is integrated into a research project on the assessment of the quality of life of the population in extreme poverty in a municipality in the RMBH. In order to undertake this, all the ethical criteria found in the National Health Council Resolution N.466, 2012, were respected. The project was approved by the Research Ethics Committee, under Opinion N. 188,866. The collaboration of the municipal departments was established with the signing of the Institutional Co-participation Declaration. All interviewees' anonymity was preserved. The profile of the multi-professional team is presented in Figure 1, identifying them by numbers and acronyms referent to the Municipal Health Department (SMS), Social Work Department (SMAS) and Education Department (SME), in which they work. For example: "7^th^ subject from Health" (7 SMS).

*General Management; †Central Technical Reference; ‡ Family Health Strategy.

## Results

The sociodemographic profile of the multi-professional team: of the 27 interviewees (Figure 1), 11 worked in the SMS, 11 in the SMAS, and 5 in the SME. A total of 7 of them were educated to senior high school level, 20 had degrees (5 SMS; 10 SMAS; 5 SME), and 14 had *latu-sensu* postgraduate qualifications (5 SMS; 5 SMAS, 4 SME). The mean age was 35 years old; the oldest person was 63, and the youngest, 25 years old. A total of 16 lived in other municipalities of the RMBH.

Five nurses and one nutritionist had worked in positions in the SMS, on average, for one year and eight months; the Community Health Workers (ACS) had worked for 11 years and one month; the others from the SMAS had worked for three years and one month, and those from the SME had worked for six years and six months. Among those with degrees, nine (1 SMS; 5 SMAS; 3 SME) and one educated to senior high school level (SMAS) worked under the statutory arrangement, and four worked in statutory commissioned positions (1 SMS; 1 SMAS; 2 SME). A total of 17 worked under employment links with temporary contracts (10 SMS; 6 SMAS; 1 SME), of whom four worked in commissioned positions (1 SMS; 2 SMAS; 1 SME).

The interviewees' knowledge regarding the principal barriers stopping the families in extreme poverty from benefiting from universal health coverage and universal access to health are described below. PAHO's 4 Strategic Lines, considered indispensable for reorienting the public policies towards universal health coverage through the SUS, encompassed the thematic axes. 1^st^ Strategic Line: expansion of fair access to the integral health services with person-centered quality.

*There is a hopelessness on the part of the population: I won't go to the clinic, it won't resolve anything, it won't work, they won't attend me, there aren't any doctors. This infrastructure, in general, leads to despondency: there is never anybody there to attend you, so will there be today? There is no point taking me there, I can't manage to get a consultation... Am I likely to get one now?* (27 SME).

*The majority complain about poor health, in spite of there being 50 or so Family Health Programs (PSF), the teams are understaffed [...] There isn't a doctor [...] and it is difficult to arrange a consultation with a specialist* (18 SMAS).

*There are shortcomings in many areas [...] Primary Care could help, help in the community. This is not just a health problem, it's much bigger, it's about infrastructure, lack of links, absence of strengthening of the network* (1 SMS).

*When I came to the PSF, the Community Health Workers would say: we have been abandoned. They would sit on the rubbish bins, they had nothing, absolutely, nothing, do you understand what I mean by 'nothing'? Everything here has been donated, these chairs and the other furniture in our PSF and others. [...] I said, I will get somebody who is doing community service to rip out those rotten wooden floortiles, I received donations and we changed the floor. I became tired of asking for help from the City Council and nobody doing anything. Over there, there used to be a brick-built septic tank, which, one day, collapsed! The ground fell in! We didn't know whether to receive patients or dispense medications, I was watching a boy playing there in the middle, or to telephone for the department... They didn't do anything there! I said to them: I deeply respect your work, but mine also needs to be respected, won't anybody do something? I will telephone health surveillance for them to close this over. I'm going to the health surveillance people now, if you won't do something. [...] And then the person responsible appeared... What public authority is there, that we can count on?* (5 SMS).

The absence of a multidisciplinary approach to the problems, in the attendance to these families' health and needs, ends up exceeding the PSF's resolutive capacity

*These families are invisible, they are nowhere: they are only in that place where they are living, they are not going to be registered in the PSF, they are not going to be registered with the Center of Reference in Social Work* (CRAS), *they are not going to be registered on the Family Allowance Program; they are there, but in the eyes of the governmental bodies and their facilities, they don't exist...* (15 SMAS). 

*In this neighborhood, there are 1,600 unregistered families, dissatisfied that they are living there, there is no school, there's no healthcare, there is no PSF and there are no buses [...] I don't know all the neighborhoods, there are various registered by the Basic Reference Units* (UBR), *but these families are not monitored!* (19 SMS). *The* PSF *is much closer to these families than the other health bodies or departments, it is a center of reference; but it cannot do everything, it doesn't have the conditions to carry out the role of the* CRAS*. There is a certain limitation, there is a space where it can act, and another where it cannot. There is a tendency to throw more things to the PSF, but the conditions are not provided to do what they already are doing, and less still to undertake anything else which might come along! The managers have a lot of difficulty promoting interdepartmental actions! If you don't do it in an integrated way and empower people, things don't work! If the work was interlinked, it would have greater resolutive power. It is necessary to provide working conditions, and to know what facilities the network has available; but people don't know about the viable facilities! The institution itself doesn't create them and does not seek to interlink them! If the institutions' missions were defined, it would be easier!* (3 SMS).

2^nd^ Strategic line: strengthening of the management and governance. The public policies and actions currently in place in territories of extreme poverty must be structured and planned in a contextualized way, so as to favor universal health coverage and universal access to health. 

*Before, the population of areas X and Y was attended by the same PSF. Then they separated the PSFs into area X and area Y, but the areas were badly divided. Those who worked and lived in X are now given consultations only in Y. What sense does that make if she belongs to area X? Whoever made this division didn't look at the area to find out about the micro-areas... This has already been put to the department and the coordinators. When this division took place, and the PSF was separated by areas X and Y, it was terrible! All the windows were broken! The population did not accept it...* (5 SMS).

*There is a lot of respect for the professionals, even when they are circulating in areas where there is a lot of conflict because of the drugs trade... The school only works with the permission of the drugs trade, as it is located in the territory of great social vulnerability. This is the system which these people live with. [...] This oppression all the time [...]... It is very, very difficult!* (26 SME). *In the city, there are the PSFs and the UBRs. The* UBR *is like a clinic for people who don't have a PSF. In this area, there is the PSF, but if you don't live in the area of the PSF, there is no clinic. The* UBR *is the clinic for those who are not registered at the PSF. These people go to the UBR, which attends about 50,000 people. The PSF's coverage is very low!* (14 SMS).

*They took her to the Emergency Room and nobody knew what was happening. The doctor said: this is negligence on the part of the municipality! Negligence regarding the community, which is there to be attended. If she has diabetes and all these health problems, she needs to be monitored! There is no doctor here, but there is the health department to provide assistance and specialities which do not exist, and which need to be arranged in another place, as they are not available here, and have to be arranged in another locale, if one is to have the privilege of this attendance! They have lost sight of what is important... ! The respect for rights, which should be offered!* (27 SME).

The social participation of the population in extreme poverty and of the health professionals is essential if one is to re-elaborate public policies which are coherent and sustainable in the long term, in these territories, and directed towards the resolution of their principal health problems. *Professionals establish a bond? There's no way to do that! For the programs of that department, the bond* (with the population) *exists and has to exist... When the person is welcomed, and embracement is present, she goes back to work saying that she was attended well, and creates a reference: you can go to so-and-so, in such-and-such a place!* (25 SME).

*The operative groups used to take place before the PSF changed place. The house* (rented) *was very bad, so the PSF was deactivated for a while, and now it has moved here! This has affected things enormously! It is very difficult for the population to get here. The neighborhood is big, they only come here when there is nothing else for it! Now, the population has a lot of difficulty! The PSF is too far away! There is transport, but not everybody has the money to pay for it. The price of the bus ticket is R$2.65. The bus used to pass close by the PSF, and the people used it. Nowadays, if they need to come to the PSF, the bus doesn't come here anymore! They need to get another bus which goes into the city center, and that costs them R$4.10. They complain, but...* (11 SMS).

*If the person is dying, I say: no, go to the PSF! Here, there is no equipment, the doctor can't do anything, and there are no drugs for the assistance! [...] When there is an emergency situation in the PSF, we call the Emergency Care Units* (UPA). *The nurse provides the attendance, blood pressure, pulse, and sends the patient to whichever UPA has a doctor. There is no ambulance to take them, they have to take care of that themselves! Police, only if you can manage it! The population doesn't always have vehicles. It is a difficulty! You can only get health transport if you arrange it 15 days beforehand!* (10 SMS).

For the elaboration of public policies, it is necessary to include the evaluation of the availability of the local technologies in this process, thus favoring possible improvements in coverage and access. *The service users who are not attended in the PSF overload the UPA! We do not have the ideal for the population's health! The new medical staff promised under the "Mais Médicos"* ["More Doctors"]* *program have arrived. We will see if they will meet all of the population's needs! [...] She complains a lot about this abandonment, about the expense and the strain when they need to do tests..* (22 SMAS).

*The PSF should treat what is basic, and have a general practitioner, a gynecologist and a pediatrician. There are three places available a month, but I have 20 children on the books! When will the last consultation be? For a gynecological issue involving infection, you have to wait more than 60 days to be seen!* (10 SMS).

*We spent more than one year without having the prevention Kit. Now, there are no specimen pots! There is no pediatrics service, but there are 95 children; we have been waiting for cardiology for two years; for orthopedics, there are more than 40 people in the queue; a gastric specialist only came because we took legal action* (5 SMS).

3^rd^ Strategic line: an increase and improvement in the financing, promotion of equality, efficiency and elimination of direct payment on the part of the service users. 

*Today, there were two children for the pediatric service, but they had been referred to "X Center". These two twins have sickle-cell anemia. I requested an urgent blood test... The parents didn't have the money to take them there to do the test, to take the bus! The test was authorized, but, due to the difficulties caused by their low purchasing power... [...] The PSF has 15 places for tests in the UBR; but in this case, it was a test which needed to be processed urgently! There was no point taking the blood sample here, we would have waited one week to get the result...* (3 SMS).

4^th^ Strategic line: Strengthening of the interdepartmental actions in order to address the social determinants of health. It is essential to carry out diagnoses in order to promote viable mechanisms for confronting the local priority problems in relation to access. 

*It is impossible to provide care in the long-term in the PSF, its structure does not allow this! [...] How wonderful it would be if they could deal with this stream, put a structure over it, a bridge, so that the children could go over it and get to school!* (10 SMS).

*The PSF's structure is very poor, and too far away! We haven't been able to put together the basic team to provide attendance... There are various PSFs without doctors or nurses. It becomes difficult!! It is impossible to provide high complexity care. Many service users have abandoned basic treatment for hypertension and diabetes! And the management, what do they understand of this?* (21 SMAS).

*We try to work more broadly with the families, referring them to the CRAS, but there is no referral or counter referral, no joint action with the health service. The public policies are piecemeal! Teams, infrastructure and transport are missing. We tried to articulate with health in the schools; this does not resolve the problem of students with priority for health reports, because there is still a lack of transport for them to go to the consultations!* (24 SME).

The interdepartmental public policies aim to strengthen the social programs and to improve these families' health conditions and living conditions; their effects must be assessed. 

*In order to improve, it's necessary to work on interdepartmentality and to investigate other instruments* (23 SME).

*If you have a child whose weight is low, you have recourse to the Child's Health Program, to the low weight department, vitamins, and the low-weight diet. However, the population does not benefit. It has to be directed towards a specific type of bond, because the PSF provides a general attendance. [...] You're not going to succeed in resolving the cause!* (10 SMS). It is the City Council's job to structure the public policies and contextualize them, and to promote progressive improvements in the health and quality of life of extremely poor families, contributing to their autonomy and to the promotion and protection of their health over time. 

*If a person is coming to the center a lot, I go without lunch and say this: today, I am for so-and-so! I dedicate myself to the patient, I listen to them carefully, I don't stop to write things down, and when she leaves, I write it in the medical records. That way, she tells me everything in front of me! And then I can see why her hypertension is not controlled, her diabetes is not controlled, and the doctor just increasing the medications and antidepressants. I see the entire context which surrounds this family, and then I can resolve the issue which is not controlled...* (4 SMS).

*In a situation of extreme poverty, you can't access anything! They are ill, depressed!* (18 SMS).

*Here, the policies and the management needs to improve in everything! The politicians come into power, t hey are in the job for four years, and then another one comes who doesn't know about the conditions here [...] The only thing they like to do here is prisons!* (7 SMS).

*What does it mean to be a citizen? The people don't know, they haven't had access to health, culture or education! This needs to be presented if they are to be able to make choices! These were the choices which they had...* (4 SMAS).

*Continuous attendance does not exist in the PSF... Nowadays, all the departments are relying on the PSF, like they're on top of a little ant. The spontaneous demand can swallow any PSF! We try to plan, but are unable to promote health... There should be more teams, so that each could have a smaller population. The quality of the work would be better!* (4 SMS). 

*Health is the most department which suffers most from governmental cuts; however, if there is no health, there is no life, and no way of getting anything!* (3 SMS).

## Discussion

The universal health coverage provided through the APS in the municipality studied has the capacity to attend only half of the areas covered and of the local population. These subjects do not represent the families in extreme poverty in the region, who are described as *invisible*; it is estimated that the number of these subjects is probably far higher. The Constitution of the World Health Organization (WHO), the PAHO guidelines and the organic laws of the SUS aim to promote improvements in the population's health, with emphasis on groups with the greatest social vulnerability^(^ 1 ^)^. In the context studied, there is a gap in relation to the PAHO guidelines in line with the actions of the SUS, leading to barriers which, for families in extreme poverty attempting to access healthcare holistically, are unsurpassable. These barriers result from failures in the process of mediation, implementation, assessment and re-elaboration of the local public policies. 

The access to health actions denied to these families who are subjected to intense social inequalities reinforces their lack of access to the other opportunities for quality of life. The inadequate management and governance of the local public policies - associated with the inconsistent application of financial resources, lack of equipment and sufficient material and human resources for implanting health actions and interdepartmental actions capable of resolving issues in the APS - represent the principal barriers to universal health coverage and universal access to health for these subjects. These data are corroborated by other studies emphasizing that increasing the APS's capacity for offering services can maximize the responses to the priority requirements in territories of poverty^(^ 9 ^-^ 10 ^)^. 

The profile and the accounts of the multi-professional team, described in this study, point to the existence of poor working conditions, and weak employment links of the majority, who work under temporary contracts. This context favors the discontinuity of the actions in the public services network and, in particular, in health, over time, the breaking of the bonds established with the families in these territories, and the devalorization of the knowledge and experiences of the team which are necessary if improvements have to be provided in the attendance to the needs of these groups, resulting from the absence of their participation in the processes of elaboration of the public policies. 

This disinterest on the part of the municipal management in providing these people with a voice in the decision-making processes and implementation of the priority health actions in each micro-region to meet the requirements of the population in extreme poverty, in a hierarchical and resolutive way, in order of importance, maximizing the APS's capacity, causes severe consequences in the planning and execution of the local public policies, and culminates in barriers which, for these families, are impossible to overcome if they are to access their rights to health and social rights, such as education, quality-of-life, social inclusion, productive inclusion, citizenship and autonomy.

These issues also reveal the absence of a prior local diagnosis in order to elaborate, implement and assess the health actions in the APS, and signify a mismatch in the light of the urgent need to reverse the adversities in relation to the social inequality. This scenario contributes to the imprisonment of these families in a repetitive lifecycle, characterized by disadvantages in access to health and to the rights to citizenship^(^ 9 ^,^ 11 ^)^. Studies carried out by the United Nations on the offering of integral health services through the APS, in Latin America, Brazil, South Africa, Ethiopia and other countries consider that, if there is to be equal health attendance, it is necessary to act on the social determinants, seeking responses which foster progress in the models of (re-)structuring in the health systems^(^ 11 ^-^ 15 ^)^. When the offering of services is limited due to failures in the coverage, the team's work is made difficult, as is the access to health, leading to the APS having a poor resolutive capacity^(^ 13 ^)^.

In these territories of extreme poverty, it is essential to undertake health actions which are consistent and endowed with social responsibility and sensitivity to valuing the local problems; and to adopting measures which are sustainable in the long term for confronting these with resolutive capacity. Aspects such as the poor urban infrastructure of the public services in these territories, local violence resulting from the drugs trade, absence of accessible public transport and lack of professionals and equipment for the continuity of the care are social determinants which neglect these groups' universal access to health, causing them hopelessness in relation to the attendance offered by the public services, discouragement to seek assistance, and failure in adherence to - or abandonment of - the treatments and care measures instituted. 

This study identifies the collective social responsibility established by the multi-professional team in response to the urgent need to promote an effort integrating all the departments in resolving the problems indicated, as suggested in the scientific literature^(^ 9 ^-^ 15 ^)^. It is important to undertake further research - testing possible tools for applying in local diagnosis with extremely poor communities, addressing multidimensional instruments for evaluating the obstacles to, and the gains obtained in, universal health coverage and universal access to health - making it possible to indicate sustainable paths for maximizing the resolutive capacity of the APS and ESF in these territories. There is, however, a shortage of such studies in Brazil and worldwide^(^ 15 ^-^ 17 ^)^.

The multi-professional team responsible for the municipal public services, in dealing with problems arising from these social inequalities, both knows and experiences in its routine the health needs of these communities in extreme poverty. Among the managers and professionals interviewed, nursing stands out due to its in-depth work and knowledge regarding the barriers which these families suffer in relation to universal health coverage and universal access to health. 

The nurses described the undertaking of actions which are committed to confronting the social inequalities experienced in these territories when they promote person-centered and humanized embracement, viabilize integral health care, provide improvements in the infrastructure of the PSFs, and minimize the local barriers to equal access to health. Nursing is an important leadership in the APS for the territories in extreme poverty; they are managers and care professionals who work in various processes reflecting on and promoting health actions and comprehensive care in this context, and present relevant evidence for the assessment, reconstruction and reorientation of the SUS's public policies in Brazil. Their perspective and work in the APS represent a social practice which is respectful, attentive and warm-hearted, understanding and politically-engaged with the adversities suffered by these groups. It is essential to value Nursing's perspective regarding the social determinants of health in these territories of extreme poverty, as a legitimate process for obtaining essential support for confronting the causes of the disparities in universal health coverage and universal access to health - and to the social rights which are denied to these families. 

## Conclusion

Privation in the access to health rights of families in extreme poverty impedes them from achieving or making proper use of universal health coverage or universal access to health. This worsens their social exclusion and spatial segregation and the social determinants of health in these territories. The principal barriers to this access are; shortcomings in the expansion and strengthening of the APS; absence of diagnosis of priority needs; shortage of technology and material and human resources; poor infrastructure of the services; APS with low resolutive capacity, and absence of interdepartmental public policies. Within the multiprofessional team, Nursing stands out as it acts in these territories with an indepth view, adopting social practices which are unique and committed regarding the problems of coverage and fair access to health. This knowledge provides nursing with support which is essential for fostering the processes inherent to public policies geared towards the re-orientation of the SUS and towards fairness in the right to health. 

## Footnotes

* The "*Mais Médicos*" ("More Doctors") program is a governmental program which involved increasing the number of medical staff. Translator's note.
